# Treatment patterns in patients with age-related macular degeneration and diabetic macular edema: A real-world claims analysis in Dubai

**DOI:** 10.1371/journal.pone.0254569

**Published:** 2021-07-13

**Authors:** Igor Kozak, Avinash Gurbaxani, Ammar Safar, Prasan Rao, Amal Masalmeh, Hazar Assaf, Mohamed Farghaly, Prathamesh Pathak, Ashok Natarajan, Insaf Saffar

**Affiliations:** 1 Moorfields Eye Hospitals, Abu Dhabi, United Arab Emirates; 2 Medcare Eye Centre, Dubai, United Arab Emirates; 3 Department of Ophthalmology, Novartis Middle East FZE, Dubai, United Arab Emirates; 4 Dubai Health Insurance Corporation, Dubai Health Authority, Dubai, United Arab Emirates; 5 IQVIA AG, Dubai, United Arab Emirates; 6 Novartis Pharma AG, Basel, Switzerland; University of Oxford, UNITED KINGDOM

## Abstract

**Objectives:**

To characterize the pattern of approved anti-vascular endothelial growth factor (VEGF) treatments among patients with neovascular age-related macular degeneration (nAMD) and diabetic macular edema (DME) in the United Arab Emirates (UAE).

**Method:**

This was a retrospective, nonrandomized, observational cohort analysis of the Dubai Real-world Claims Database with a 360-day follow-up period. Adult patients diagnosed with nAMD or DME treated with ranibizumab or aflibercept for the first time were included. The primary objective was to evaluate anti-VEGF treatment patterns with respect to the proportion of patients receiving ranibizumab and aflibercept for nAMD and DME separately.

**Results:**

Of the 451 patients included in the final study cohort, 83.6% and 16.4% had a diagnosis of DME (ranibizumab: 48.5%; aflibercept: 51.5%) and nAMD (ranibizumab: 40.5%; aflibercept: 59.5%), respectively, at baseline. Treatment frequency of ranibizumab/aflibercept was similar for nAMD (mean: 2.4/2.9 injections; p = 0.2389) with fewer injections in the ranibizumab cohort for DME (mean: 1.9/2.5 injections; p = 0.0002). Most patients received ≤3 anti-VEGF injections during the 360-day follow-up period. The time between consecutive treatments was large (nAMD: 73.6 days/10.5 weeks; DME: 80.5 days/11.5 weeks). Approximately 10%–13.5% of patients switched their anti-VEGF therapy. Most patients (83.8%) had a diabetes diagnosis during the follow-up period.

**Conclusions:**

This real-world study provides an initial understanding of anti-VEGF treatment patterns in patients with nAMD and DME in the UAE. Treatment frequency of the 2 anti-VEGF agents assessed was similar in both patient populations. Both treatments were infrequently administered with large dosing intervals.

## Introduction

Age-related macular degeneration (AMD) and diabetic retinopathy (DR) are two of the leading causes of visual impairment worldwide [1]. Diabetic macular edema (DME) left untreated is the primary cause of vision loss in patients with DR [2]. The projected global prevalence of neovascular AMD (nAMD) in 2040 is 288 million [3]. There are approximately 21 million people with DME worldwide [4]. DR and DME affect approximately 26% and 9% of patients with diabetes in the United Arab Emirates (UAE), respectively [5].

Anti-vascular endothelial growth factor (anti-VEGF) therapy is the standard of care for the management of nAMD and DME [6, 7]. Ranibizumab was approved for the treatment of nAMD (Food and Drug Administration [FDA]: 2006; European Union [EU]: 2007) and visual impairment due to DME (FDA: 2012; EU: 2011) [8, 9]. Aflibercept was also approved for the treatment of nAMD (FDA: 2011; EU: 2012) and visual impairment due to DME (FDA and EU: 2014) [10, 11]. The efficacy and safety of ranibizumab and aflibercept in the treatment of nAMD and visual impairment due to DME are well documented [12–19]. Both ranibizumab 0.5 mg and aflibercept 2.0 mg have been approved and available for the treatment of nAMD and DME in the UAE from 2007 and 2014, respectively.

Intravitreal anti-VEGF injection treatment warrants frequent monitoring of disease activity, number of injections, follow-up visits, and dosing intervals [20]. The analysis of the current anti-VEGF real-world treatment patterns helps to gain an understanding of such challenges for better disease management and patient follow-up. Real-world data on the treatment outcomes of nAMD and DME in the UAE is nonexistent. The current study observed the treatment patterns in patients with nAMD and DME managed with ranibizumab or aflibercept using the Dubai Real-world Claims Database (DRWD).

## Materials and methods

### Study design

This study was a retrospective, non-comparative, non-randomized, observational cohort analysis that assessed the treatment patterns of ranibizumab and aflibercept in patients with nAMD and DME in Dubai, UAE.

Data were collected from the DRWD, an anonymized longitudinal patient-level database of insurance claims generated from the private healthcare sector in the Emirate of Dubai (<0.1% of the total claims were from the public sector).

The study period was from January 2014 to February 2019, with a patient screening period between July 2014 and February 2018; patients who received anti-VEGF after November 01 2014 were included. This allowed for the collection of data with 10–12 months before the first anti-VEGF injection and a 360-day follow-up period within the database.

### Patient population

This study was conducted separately for nAMD and DME. Patients were categorized into the ranibizumab or aflibercept cohort depending on the treatment received on the date of the first anti-VEGF injection. Adults diagnosed with nAMD or DME who were treated with ranibizumab or aflibercept for the first time were included.

Patients had to fulfill the following characteristics: (1) at least 1 claim with a diagnosis of nAMD or DME, defined by the International Classification of Diseases, 10th Revision, within the screening period; (2) receipt of ranibizumab or aflibercept on or after diagnosis and after November 01, 2014; and (3) a minimum follow-up period of 360 days from the screening date, defined based on the activity in the database for which patients needed at least 1 claim related to any activity during the first and second 6 months after the first injection. The exclusion criteria are listed in **S1 Appendix.**

### Study objectives and endpoints

The main objective was to understand the pattern of receipt of the approved anti-VEGF treatments among patients with nAMD and DME in the UAE. The primary endpoint was to evaluate the number and proportion of patients with nAMD and DME receiving ranibizumab and aflibercept as their first anti-VEGF therapy.

Secondary endpoints included (1) treatment patterns, including number of anti-VEGF injections (during hospital visits, outpatient visits, emergency department visits, and others) received during the first year of treatment and the time from the date of diagnosis of nAMD or DME to the date of the first anti-VEGF treatment (time to treatment);(2) switch in anti-VEGF therapy, including number and proportion of patients who switched at Month 12 and the mean switch time at Month 12.

Exploratory endpoints were (1) diabetes endpoints, which included the number and proportion of patients with type 2 diabetes mellitus; the diagnosis and use of oral anti-diabetic drugs (OADs) and insulin, including frequency of prescriptions, during the follow-up period at Month 12 and medication adherence for a subset of patients with diabetes with at least 3 months of follow-up from the diabetes treatment date and (2) endpoints for potential bilateral patients, including the number of patients receiving 2 or more injections of ranibizumab or aflibercept on the same day or receiving 2 injections within a period of 21 days and the use of OADs and insulin, including frequency of prescriptions, among the potential bilateral patients.

### Study assessments

Baseline patient characteristics were assessed during the 10–12-month period prior to the first anti-VEGF injection date, and treatment patterns (injection frequency, dosing intervals, administration setting, and switching) were assessed during the 360-day period following the date of the first anti-VEGF injection. Demographic and clinical characteristics collected included age, gender, geographic location, and the Charlson comorbidity index (CCI). Anti-VEGF injection patterns, including the number of anti-VEGF injections in all settings (inpatient, outpatient, emergency department, and other settings) during the first 12 months and time from diagnosis to the first anti-VEGF treatment, were assessed (details of the study assessment are provided in **S2 Appendix**).

Since the study protocol involves the collection and analysis of secondary data from the DRWD, an anonymized longitudinal patient-level database, and the core study proposed herein does not involve the collection, use, or transmittal of individual identifiable data, the study did not warrant an ethics committee/institutional review board approval as advised by the Dubai Scientific Research Ethics Committee (Medical Education and Research Department, Dubai Health Authority), after protocol submission. The study was conducted in accordance with the tenets of the Declaration of Helsinki. All patient identifiers were protected according to the Health Insurance Portability and Accountability Act. Patient identifiers were stripped out completely. On this basis, a formal ethics committee approval was not required, and written informed consent was not sought for this study.

### Statistical analysis

Descriptive statistics were reported. The analyses were conducted for study patients with at least 12 months of follow-up. Follow-up was defined based on activity (at least 1 claim) during each 6-month period. Continuous variables were summarized by providing the number of observations, means, and standard deviation (SD). Categorical variables were summarized by providing counts and proportions, with missing data considered as a separate category. Although this study reported descriptive analysis, the difference between the ranibizumab and aflibercept cohorts in the number of injections and time to first injection was analyzed post-hoc using a 2-sample t-test with p < 0.05 to define significant difference. All analyses were performed by IQVIA, Dubai, using the Statistical Analysis System (SAS) 9.4 software.

## Results

A feasibility analysis showed that the DRWD has 4,723 and 300 patients with first diagnosis claims for DME and nAMD, respectively, between July 2014 to April 2017 (details of the feasibility analysis are provided in **S3 Appendix**).

**Fig 1:**
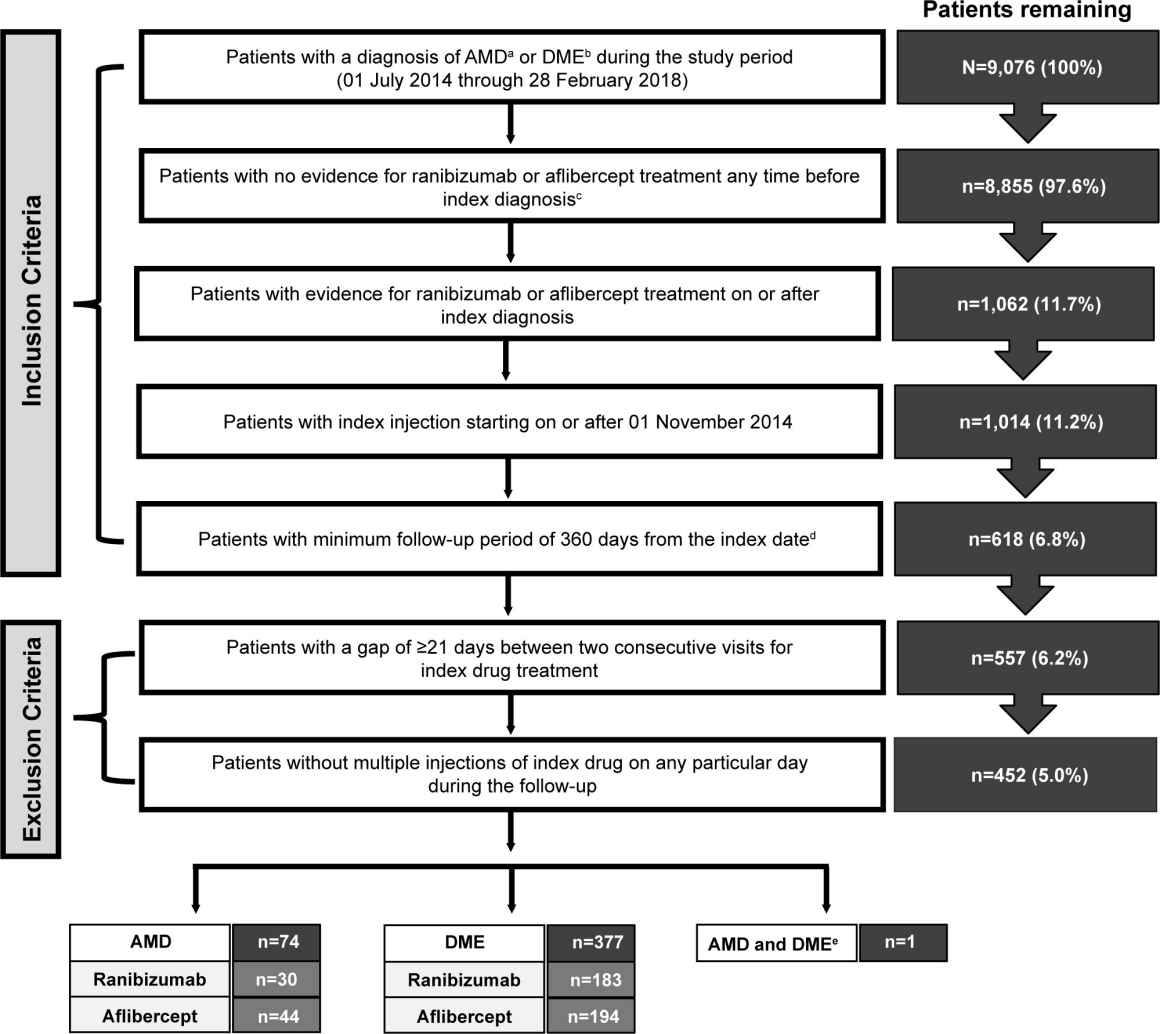
Patient selection criteria and attrition of study population. ^a^ICD-10 diagnosis codes for AMD: H35.32. ^b^ICD-10 diagnosis codes for DME: E11.311/21/31/41/51, E13.351, E08.341, E08.311. ^c^Index diagnosis: The date of first diagnosis of AMD or DME. ^d^Index date: The date of first treatment with ranibizumab or aflibercept. ^e^One patient was diagnosed with both AMD and DME and was treated with aflibercept. AMD, age-related macular degeneration; DME; diabetic macular edema; ICD-10, International Classification of Diseases, 10th Revision; N, total number of patients; n, number of patients.

Overall, there were 9,076 patients with a diagnosis of nAMD or DME from July 01, 2014, to February 28, 2018. After excluding patients based on protocol-defined inclusion and exclusion criteria, the final study population included 5.0% of patients (n = 451). There were 74 patients (16.4%) with a diagnosis of nAMD (ranibizumab: n = 30 [40.5%]; aflibercept: n = 44 [59.5%]). Most patients (n = 377 [83.6%]) in the final study population had a diagnosis of DME (ranibizumab: n = 183 [48.5%]; aflibercept: n = 194 [51.5%]) (**Fig 1**).

### Baseline demographics

A total of 74 and 377 patients were diagnosed with nAMD and DME, respectively. Demographic data were available for 17 (23.0%) and 70 (18.6%) patients with nAMD and DME, respectively. The mean (SD) age was 55 (17.1) years (ranibizumab: 51.6 [14.3] years; aflibercept: 58.0 [19.5] years) for patients with nAMD and 49.1 (12.6) years (ranibizumab: 49.7 [13.0] years; aflibercept 48.0 [12.1] years) for patients with DME. Most patients were <65 years of age (nAMD: 70.6% [ranibizumab: 62.5%; aflibercept: 77.8%]; DME: 91.4% [ranibizumab: 86.7%; aflibercept: 100%]). Gender distribution was equal among patients with nAMD, while most patients with DME were male (n = 59 [84.3%]) (**Table 1**). The mean (SD) CCI score was 1.0 (1.6) for the overall population of patients with nAMD (ranibizumab: 1.1 [1.7]; aflibercept: 0.9 [1.6]) and 2.8 (1.4) for the overall population of patients with DME (ranibizumab: 2.9 [1.3]; aflibercept: 2.7 [1.5]). In patients with nAMD, 39.2% had a CCI score of ≥1; the ranibizumab cohort had a slightly higher proportion of patients with a CCI score of ≥1 compared with the aflibercept cohort (46.7% vs 34.1%). In patients with DME, 51.5% had a CCI score of ≥3; the ranibizumab cohort had a slightly higher proportion of patients with a CCI score of ≥3 compared with the aflibercept cohort (53.6% vs 49.5%). In patients with nAMD, the ranibizumab cohort had a slightly higher proportion of patients with diabetes without complications compared with the aflibercept cohort (26.7% vs 18.2%); both cohorts had a similar proportion of patients with DME with a diagnosis of diabetes without complications (49.2% vs 50.5%). In patients with nAMD, the ranibizumab cohort had a relatively lower proportion of patients with diabetes with complications compared with the aflibercept cohort (16.7% vs 22.7%); while in patients with DME, the ranibizumab cohort had a relatively higher proportion of patients with diabetes with complications compared with the aflibercept cohort (95.1% vs 86.6%) (**Table 1**).

**Table 1:** Baseline demographic and clinical characteristics of the study patient population.

Characteristics	nAMD			DME		
	Overall	Ranibizumab	Aflibercept	Overall	Ranibizumab	Aflibercept
**Overall, N (%)**	**74 (100.0)**	**30 (100.0)**	**44 (100.0)**	**377 (100.0)**	**183 (100.0)**	**194 (100.0)**
**Gender, n (%)**						
Female	8 (10.8)	3 (10.0)	5 (11.4)	11 (2.9)	7 (3.8)	4 (2.1)
Male	9 (12.2)	5 (16.7)	4 (9.1)	59 (15.6)	38 (20.8)	21 (10.8)
Missing	57 (77.0)	22 (73.3)	35 (79.5)	307 (81.4)	138 (75.4)	169 (87.1)
**Age, years**						
n	17	8	9	70	45	25
Mean (SD)	55.0 (17.1)	51.6 (14.3)	58.0 (19.5)	49.1 (12.6)	49.7 (13.0)	48.0 (12.1)
**CCI components**^**a**^**, n (%)**						
Congestive heart failure	2 (2.7)	1 (3.3)	1 (2.3)	13 (3.4)	2 (1.1)	11 (5.7)
Peripheral vascular disease	0 (0.0)	0 (0.0)	0 (0.0)	10 (2.7)	6 (3.3)	4 (2.1)
Cerebrovascular disease	4 (5.4)	1 (3.3)	3 (6.8)	7 (1.9)	3 (1.6)	4 (2.1)
Chronic pulmonary disease	8 (10.8)	5 (16.7)	3 (6.8)	28 (7.4)	13 (7.1)	15 (7.7)
Peptic ulcer disease	1 (1.4)	1 (3.3)	0 (0.0)	10 (2.7)	7 (3.8)	3 (1.5)
Liver disease—mild	3 (4.1)	2 (6.7)	1 (2.3)	18 (4.8)	10 (5.5)	8 (4.1)
Diabetes without complications	16 (21.6)	8 (26.7)	8 (18.2)	188 (49.9)	90 (49.2)	98 (50.5)
Diabetes with complications	15 (20.3)	5 (16.7)	10 (22.7)	342 (90.7)	174 (95.1)	168 (86.6)
Renal disease	2 (2.7)	1 (3.3)	1 (2.3)	29 (7.7)	14 (7.7)	15 (7.7)
**CCI score category, n (%)**						
0	45 (60.8)	16 (53.3)	29 (65.9)	24 (6.4)	6 (3.3)	18 (9.3)
1–2	20 (27.0)	11 (36.7)	9 (20.5)	159 (42.2)	79 (43.2)	80 (41.2)
3–4	7 (9.5)	2 (6.7)	5 (11.4)	157 (41.6)	80 (43.7)	77 (39.7)
5–6	0 (0.0)	0 (0.0)	0 (0.0)	29 (7.7)	16 (8.7)	13 (6.7)
7+	2 (2.7)	1 (3.3)	1 (2.3)	8 (2.1)	2 (1.1)	6 (3.1)
**CCI score**						
n	74	30	44	377	183	194
Mean (SD)	1.0 (1.6)	1.1 (1.7)	0.9 (1.6)	2.8 (1.4)	2.9 (1.3)	2.7 (1.5)
**Index drug strength, n (%)**						
Ranibizumab 10 mg/mL	30 (100.0)			183 (100.0)		
Aflibercept 40 mg/mL	44 (100.0)			194 (100.0)		

^a^CCI components of ≥2% in the proportion in the overall patients with either nAMD or DME are included here.

CCI, Charlson comorbidity index; DME, diabetic macular edema; N, total number of patients; n, number of patients;

nAMD, neovascular age-related macular degeneration; SD, standard deviation.

### Treatment patterns

Overall, 153 patients with nAMD (ranibizumab: n = 76; aflibercept: n = 77) and 905 patients with DME (ranibizumab: n = 453; aflibercept: n = 452) received anti-VEGF treatment. For both patients with nAMD and DME, the mean (SD) time to primary anti-VEGF treatment was similar between both cohorts (nAMD, ranibizumab: 47.6 [107.1] days; aflibercept: 49.3 [123.2] days; DME, ranibizumab: 67.3 [140.6] days; aflibercept: 73.4 [144.4] days) (**Table 2**).

**Table 2:** Anti-VEGF treatment pattern after diagnosis.

	Overall	nAMD	DME	nAMD and DME
**Overall, n (%)**^a^	**8,855 (100.0)**	**406 (100.0)**	**8,436 (100.0)**	**13 (100.0)**
Ranibizumab	530 (6.0)	76 (18.7)	453 (5.4)	1 (7.7)
Aflibercept	532 (6.0)	77 (19.0)	452 (5.4)	3 (23.1)
No treatment	7,793 (88.0)	253 (62.3)	7,531 (89.3)	9 (69.2)
**Time to primary treatment, days** ^b^				
**Ranibizumab**				
n	530	76	453	1
Mean (SD)	64.4 (36.3)	47.6 (107.1)	67.3 (140.6)	4.0 (0.0)
**Aflibercept**				
n	532	77	452	3
Mean (SD)	69.8 (141.3)	49.3 (123.2)	73.4 (144.4)	52.0 (76.5)
**Potential bilateral patients**				
Patients with 2 consecutive visits for primary anti-VEGF treatment within a period of 21 days, n (%)^c^	61 (100.0)	4 (6.6)	57 (93.4)	0 (0.0)
Patients with multiple injections of primary anti-VEGF treatment on any particular day during the follow-up, n (%)^d^	105 (100.0)	16 (15.2)	89 (84.8)	0 (0.0)

^a^Patients with no evidence for ranibizumab or aflibercept treatment at any time before index diagnosis (n = 8855).

^b^Patients with evidence for ranibizumab or aflibercept treatment on or after index diagnosis (n = 1062).

^c^Per total number of patients with 2 consecutive visits for primary anti-VEGF treatment within a period of 21 days.

^d^Per total number of patients with multiple injections of primary anti-VEGF treatment on any particular day during the follow-up.

DME, diabetic macular edema; N, total number of patients; n, number of patients; nAMD, neovascular age-related macular degeneration;

SD, standard deviation; VEGF, vascular endothelial growth factor.

In the final study population, the number of mean (SD) primary anti-VEGF injections received by patients with nAMD (ranibizumab: n = 30; aflibercept: n = 44) and DME (ranibizumab: n = 183; aflibercept: n = 194) was 2.7 (1.7) and 2.2 (1.5), respectively, over the 360-day follow-up period (**Table 3**). Patients with nAMD in both cohorts received a similar mean (SD) number of injections (2.4 [1.4] vs 2.9 [1.9], respectively; p = 0.2389), while patients with DME in the ranibizumab cohort received a lower mean number of injections compared with those in the aflibercept cohort (1.9 [1.2] vs 2.5 [1.7]; p = 0.0002). The proportion of patients receiving ≤3 injections was higher in the ranibizumab cohort compared with the aflibercept cohort in both patients with nAMD (n = 27 [90.0%] vs n = 29 [65.9%]) and DME (n = 162 [88.5%] vs n = 150 [77.3%]). Most patients received only 1 injection during the first 30 days of follow-up (nAMD: n = 61, 82.4% [ranibizumab: 76.7%; aflibercept: 86.4%]; DME: n = 346, 91.8% [ranibizumab: 95.1%; aflibercept: 88.7%]). Patients with nAMD received anti-VEGF therapy within approximately 2 months (Mean [SD]: 57.4 [129.0] days) of diagnosis, with patients in the ranibizumab cohort receiving treatment earlier than those in the aflibercept cohort (45.2 [70.4] vs 65.6 [157.2] days; p = 0.4522). Patients with DME received anti-VEGF therapy within approximately 3 months (Mean [SD]: 94.0 [180.8] days) of diagnosis (ranibizumab: 92.1 [181.6] days; aflibercept: 95.9 [180.6] days; p = 0.8392). The mean (SD) time between 2 consecutive anti-VEGF injections was 73.6 (64.4) days and 80.5 (67.3) days for patients with nAMD and DME, respectively, and was shorter in the ranibizumab cohort compared with the aflibercept cohort in patients with nAMD (53.6 [52.3] vs 87.4 [69.1] days) and similar between the cohorts in patients with DME (77.9 [65.0] vs 82.5 [69.2] days, respectively) (**Table 3**). Most treatments were administered in other settings in patients with nAMD (71.5%) and DME (60.4%) alike (**Table 3**).

**Table 3:** Treatment pattern of primary anti-VEGF treatment during follow-up (Year 1).

Criteria	nAMD			DME		
	Overall	Ranibizumab	Aflibercept	Overall	Ranibizumab	Aflibercept
**Overall, N (%)**	**74 (100)**	**30 (100)**	**44 (100)**	**377 (100)**	**183 (100)**	**194 (100)**
**Number of primary anti-VEGF injections during follow-up, n (%)**						
1	23 (31.1)	8 (26.7)	15 (34.1)	166 (44.0)	91 (49.7)	75 (38.7)
2	16 (21.6)	8 (26.7)	8 (18.2)	79 (21.0)	40 (21.9)	39 (20.1)
3	17 (23.0)	11 (36.7)	6 (13.6)	67 (17.8)	31 (16.9)	36 (18.6)
4	7 (9.5)	2 (6.7)	5 (11.4)	32 (8.5)	15 (8.2)	17 (8.8)
5+	11 (14.9)	1 (3.3)	10 (22.7)	33 (8.8)	6 (3.3)	27 (13.9)
**Number of injections during the first 30 days, n (%)**						
1	61 (82.4)	23 (76.7)	38 (86.4)	346 (91.8)	174 (95.1)	172 (88.7)
2	13 (17.6)	7 (23.3)	6 (13.6)	31 (8.2)	9 (4.9)	22 (11.3)
**Number of anti-VEGF injections**						
n	74	30	44	377	183	194
Mean (SD)^a^	2.7 (1.7)	2.4 (1.4)	2.9 (1.9)	2.2 (1.5)	1.9 (1.2)	2.5 (1.7)
**Time to primary anti-VEGF treatment from diagnosis, days**						
n	74	30	44	377	183	194
Mean (SD)^b^	57.4 (129.0)	45.2 (70.4)	65.6 (157.2)	94.0 (180.8)	92.1 (181.6)	95.9 (180.6)
**Time between 2 consecutive injection visits, days**						
**All encounter types**						
n	49	20	29	210	92	118
Mean (SD)	73.6 (64.4)	53.6 (52.3)	87.4 (69.1)	80.5 (67.3)	77.9 (65.0)	82.5 (69.2)
**Outpatient visits**						
n	11	2	9	76	8	68
Mean (SD)	66.3 (37.2)	50.3 (19.5)	69.8 (40.0)	81.4 (69.5)	74.1 (72.2)	82.2 (69.7)
**Number of injection visits by place of service**						
**Total number of injection visits, n (%)**	200 (100.0)	73 (100.0)	127 (100.0)	839 (100.0)	355 (100.0)	484 (100.0)
Inpatient visits^c^	5 (2.5)	3 (4.1)	2 (1.6)	35 (4.2)	22 (6.2)	13 (2.7)
Outpatient visits^d^	52 (26.0)	9 (12.3)	43 (33.9)	296 (35.3)	35 (9.9)	261 (53.9)
Emergency department visits^e^	0 (0.0)	0 (0.0)	0 (0.0)	1 (0.1)	1 (0.3)	0 (0.0)
Other^f^	143 (71.5)	61 (83.6)	82 (64.6)	507 (60.4)	297 (83.7)	210 (43.4)
Daycare bed + no emergency room	143 (100.0)	61 (100.0)	82 (100.0)	503 (99.2)	296 (99.7)	207 (98.6)
Daycare bed + emergency room	0 (0.0)	0 (0.0)	0 (0.0)	4 (0.8)	1 (0.3)	3 (1.4)

^a^Mean (SD) number of anti-VEGF injections: nAMD, p = 0.2389 (ranibizumab: 2.4 [1.4] vs aflibercept: 2.9 [1.9]); DME, p = 0.0002 (ranibizumab: 1.9 [1.2] vs aflibercept: 2.5 [1.7]).

^b^Mean (SD) time to primary anti-VEGF treatment from diagnosis (in days): nAMD, p = 0.4522 (ranibizumab: 45.2 [70.4] days vs aflibercept: 65.6 [157.2] days); DME, p = 0.8392 (ranibizumab: 92.1 [181.6] days vs aflibercept: 95.9 [180.6] days).

Comparison using the 2-sample t-test with p < 0.05 used to define a significant difference.

^c^Encounter type: Inpatient bed + no emergency room.

^d^Encounter type: No bed + no emergency room.

^e^Encounter type: Inpatient bed + emergency room, no bed + emergency room.

^f^Encounter type: All other than those mentioned in c–e.

DME, diabetic macular edema; N, total number of patients; n, number of patients; nAMD, neovascular age-related macular degeneration;

SD, standard deviation; VEGF, vascular endothelial growth factor.

### Switched anti-VEGF treatment

Overall, 10 (13.5%) and 41 (10.9%) patients with nAMD and DME, respectively, were switched to a different anti-VEGF treatment, with most of these switches being for patients in the ranibizumab cohort (nAMD: n = 9/30 [30.0%]; DME: n = 32/183 [17.5%]) vs the aflibercept cohort (nAMD: n = 1/44 [2.3%]; DME: n = 9/194 [4.6%]). The mean (SD) time taken to switch treatments was 127.3 (88.4) days (ranibizumab: 108.6 [69.6] days; aflibercept: 296.0 [0.0] days) for patients with nAMD and 157.9 (100.8) days (ranibizumab: 153.2 [105.8] days; aflibercept: 174.8 [83.8] days) for patients with DME. Patients initially treated with ranibizumab received more mean (SD) injections when they switched to another anti-VEGF treatment compared with those treated with aflibercept (nAMD: 2.1 [1.7] vs 1.0 [0.0]; DME: 2.6 [1.6] vs 1.3 [0.7]). Most of the injections were administered in other settings for patients with nAMD (70.0%) and DME (65.3%) (**Table 4**).

**Table 4:** Treatment patterns for patients who switched anti-VEGF treatment.

Criteria	nAMD			DME		
	Overall	Ranibizumab	Aflibercept	Overall	Ranibizumab	Aflibercept
**Overall, N (%)**	**74 (100.0)**	**30 (100.0)**	**44 (100.0)**	**377 (100.0)**	**183 (100.0)**	**194 (100.0)**
**Patients who switched anti-VEGF treatment, n (%)**	10 (13.5)	9 (30.0)	1 (2.3)	41 (10.9)	32 (17.5)	9 (4.6)
**Time to switch, days**						
n	10	9	1	41	32	9
Mean (SD)	127.3 (88.4)	108.6 (69.6)	296.0 (0.0)	157.9 (100.8)	153.2 (105.8)	174.8 (83.8)
**Number of anti-VEGF injections**						
N	10	9	1	41	32	9
Mean (SD)	2.0 (1.6)	2.1 (1.7)	1.0 (0.0)	2.3 (1.6)	2.6 (1.6)	1.3 (0.7)
**Time between 2 consecutive drug injection visits, days**						
**All encounter types**						
n	4	4	0	23	22	1
Mean (SD)	47.8 (6.7)	47.8 (6.7)	0	70.3 (64.6)	69.5 (66.0)	88.5 (0.0)
**Outpatient visits**						
n	1	1	0	5	5	0
Mean (SD)	41.0 (0.0)	41.0 (0.0)	0	73.8 (99.8)	73.8 (99.8)	0
**Number of injection visits by place of service**						
**Total number of injection visits**	20 (100.0)	19 (100.0)	1 (100.0)	95 (100.0)	83 (100.0)	12 (100.0)
Inpatient visits^a^	0 (0.0)	0 (0.0)	0 (0.0)	11 (11.6)	9 (10.8)	2 (16.7)
Outpatient visits^b^	6 (30.0)	5 (26.3)	1 (100.0)	21 (22.1)	16 (19.3)	5 (41.7)
Emergency department visits^c^	0 (0.0)	0 (0.0)	0 (0.0)	1 (1.1)	1 (1.2)	0 (0.0)
Other^d^	14 (70.0)	14 (73.7)	0 (0.0)	62 (65.3)	57 (68.7)	5 (41.7)
Daycare bed + no emergency room	14 (100.0)	14 (100.0)	0 (0.0)	62 (100.0)	57 (100.0)	5 (100.0)
Daycare bed + emergency room	0 (0.0)	0 (0.0)	0 (0.0)	0 (0.0)	0 (0.0)	0 (0.0)
**Number of primary anti-VEGF injections before switch**						
n	10	9	1	41	32	9
Mean (SD)	1.8 (1.1)	1.9 (1.2)	1.0 (0.0)	1.7 (1.1)	1.8 (1.2)	1.6 (0.9)

^a^Encounter type: Inpatient bed + no emergency room.

^b^Encounter type: No bed + no emergency room.

^c^Encounter type: Inpatient bed + emergency room, no bed + emergency room.

^d^Encounter type: All other than those mentioned in a–c.

DME, diabetic macular edema; N, total number of patients; n, number of patients; nAMD, neovascular age-related macular degeneration;

SD, standard deviation; VEGF, vascular endothelial growth factor.

### Diabetes diagnosis, treatment, and medication adherence

Most patients (n = 378 [83.8%]) had type 2 diabetes mellitus during the 360 days of follow-up. Overall, 51.6% of patients had type 2 diabetes mellitus and received OADs or insulin. Among these patients, 42.1% received 5 or more OAD prescriptions and 30.3% received 5 or more insulin prescriptions during the 360 days of follow-up (**Table 5**).

**Table 5:** Diabetes diagnosis and oral treatment patterns in the follow-up period.

Criteria	Overall	nAMD	DME
**Overall, N (%)**	**451 (100.0)**	**74 (100.0)**	**377 (100.0)**
Number of patients with a diagnosis of T2DM during the 360 days of follow-up, n (%)	378 (83.8)	29 (39.2)	349 (92.6)
Number of patients with a diagnosis of T2DM only during the 360 days of follow-up, n (%)	219 (57.9)	18 (62.1)	201 (57.6)
Number of patients with a diagnosis of T2DM and oral anti-diabetic treatment during the 360 days of follow-up, n (%)	159 (42.1)	11 (37.9)	148 (42.4)
Number of patients with a diagnosis for T2DM and receiving oral anti-diabetic treatment or insulin during the 360 days of follow-up, n (%)	195 (51.6)	13 (44.8)	182 (52.1)
**PDC during 90 days after the first oral anti-diabetic treatment**			
n	159	11	148
Mean (SD)	0.95 (0.16)	1.00 (0.00)	0.94 (0.17)
**PDC category, n (%)**			
0.0–<0.2	3 (1.9)	0 (0.0)	3 (2.0)
0.2–<0.4	2 (1.3)	0 (0.0)	2 (1.4)
0.4–<0.6	0 (0.0)	0 (0.0)	0 (0.0)
0.6–<0.8	11 (6.9)	0 (0.0)	11 (7.4)
0.8–≤1.0	143 (89.9)	11 (100.0)	132 (89.2)
**PDC during 180 days after the first oral anti-diabetic treatment**			
n	159	11	148
Mean (SD)	0.88 (0.23)	0.91 (0.20)	0.88 (0.23)
**PDC category, n (%)**			
0.0–<0.2	2 (1.3)	0 (0.0)	2 (1.4)
0.2–<0.4	11 (6.9)	0 (0.0)	11 (7.4)
0.4–<0.6	12 (7.5)	2 (18.2)	10 (6.8)
0.6–<0.8	9 (5.7)	0 (0.0)	9 (6.1)
0.8–≤1.0	125 (78.6)	9 (81.8)	116 (78.4)
**Number of oral anti-diabetic prescriptions during follow-up, n (%)**			
0	41 (21.0)	10 (76.9)	31 (17.0)
1	22 (11.3)	0 (0.0)	22 (12.1)
2	19 (9.7)	2 (15.4)	17 (9.3)
3	17 (8.7)	0 (0.0)	17 (9.3)
4	14 (7.2)	0 (0.0)	14 (7.7)
5+	82 (42.1)	1 (7.7)	81 (44.5)
**Number of insulin prescriptions during follow-up, n (%)**			
0	66 (33.8)	2 (15.4)	64 (35.2)
1	19 (9.7)	1 (7.7)	18 (9.9)
2	16 (8.2)	1 (7.7)	15 (8.2)
3	15 (7.7)	2 (15.4)	13 (7.1)
4	20 (10.3)	0 (0.0)	20 (11.0)
5+	59 (30.3)	7 (53.8)	52 (28.6)

DME, diabetic macular edema; N, total number of patients; n, number of patients; nAMD, neovascular age-related macular degeneration; PDC, proportion of days covered; SD, standard deviation; T2DM, type 2 diabetes mellitus; VEGF, vascular endothelial growth factor.

Adherence to OAD treatment, measured as the proportion of days covered (PDC) during the 180 days after therapy, was high (mean PDC [SD]: 0.88 [0.23]; nAMD: 0.91 [0.20]; DME: 0.88 [0.23]), with 78.6% of patients having a PDC greater than 0.8. Similar results, but with slightly better adherence, were observed during the 90 days after the first OAD treatment (**Table 5**).

### Potential bilateral patients

Sixty-one patients were identified as potential bilateral patients, most were in the DME cohort and fewer than half (42.6%) of them did not receive an OAD prescription, while 65.6% of the patients did not receive an insulin prescription. A higher proportion of patients in the DME cohort had 3 or more OAD prescriptions (47.4%) vs the nAMD cohort (25.0%). Of the 105 patients with multiple injections of primary anti-VEGF on any particular day during the follow-up, more than half had no OAD or insulin prescription in both the nAMD and DME cohorts (55.2% and 68.6%, respectively). A higher proportion of patients with DME had 3 or more OAD prescriptions (38.2%) vs patients with nAMD (25.0%) as shown in **S1 Table**.

### Safety

This study was a retrospective cohort study based on previously collected data from the DRWD. Any causal assessments between the treatments and adverse events/reactions could not be confirmed at an individual level; hence, no systemic safety data were collected or reported.

## Discussion

To the best of our knowledge, this is the first study to characterize anti-VEGF treatment patterns among patients with nAMD and DME in Dubai, UAE, and the Middle East.

In this study, patient demographic information was not completely available, which may limit interpretation. However, we observed a trend wherein many patients with nAMD and DME were <65 years of age, which could have been due to the non-uniform reimbursement policies and population distribution coverage of the multiple insurance systems. This may account for the overall lower age reported in the current study based on the DRWD compared with the average age reported in other real-world studies [21]. A higher number of patients aged <65 years may also indicates a shift in the onset of the disease in the population, attributable to changing lifestyle and dietary habits and a mixed population including locals and expatriates within the DRWD. We observed a trend for equal distribution of male and female patients with nAMD. The gender and mean age profile at baseline in this nAMD cohort may be slightly different from that in the epidemiologic studies from the Western world [22–24].

Treatment frequency of the 2 anti-VEGF treatments assessed was similar for patients with nAMD (p = 0.24). The patients with DME in the ranibizumab cohort received a slightly lower number of injections (p = 0.0002). The findings of the current study are consistent with the results of a retrospective 12-month cohort study in the United States (US) (ranibizumab: 5.8 vs aflibercept: 5.5 injections [mean]) [25] and a retrospective claims database study in Switzerland (3.9 injections) [26]. Another US study based on a claims database also reported a similar treatment frequency between ranibizumab and aflibercept (4.9 vs 5.2, respectively) [27]. However, the treatment frequency was higher in these real-world studies in the US, possibly owing to differences in healthcare systems and greater awareness among clinicians and patients. A higher number of injections has been reported in longitudinal studies, such as LUMINOUS (ranibizumab: 5.0) [24] and the *Fight Retinal Blindness*! *(FRB*!*)* study (ranibizumab: 7.3 vs aflibercept: 7.2) [28] at 12 months.

Ranibizumab and aflibercept were infrequently administered, with most patients receiving ≤3 injections during the 360-day follow-up period, which was much lower than the recommended administration schedule for ranibizumab and aflibercept [29, 30]. In real-world studies, patients receive fewer anti-VEGF injections compared with those in randomized controlled trials [21, 24, 31–34]. The low frequency of injections may be attributable to *pro re nata* (PRN) treatment regimens to decrease the treatment burden for patients or a less strict follow-up [31].

The mean (SD) CCI score was low (1 [1.6]) in patients with nAMD, which may have correlated with the baseline mean age of 55 years for the overall population. The authors have observed that a higher CCI score is related to a greater number of injections due to more systemic comorbidities, which make these patients nonresponsive to anti-VEGF treatment, necessitating more treatment or injection visits. The mean (SD) CCI score was higher in patients with DME compared with those with nAMD, probably owing to the higher number of patients with diabetes in the DME cohort.

Patients with nAMD received anti-VEGF treatment within approximately 2 months, with patients in the ranibizumab cohort receiving the index drug injection earlier than the patients in the aflibercept cohort (45.2 days vs 65.6 days). Patients with DME received anti-VEGF treatment within approximately 3 months; minor differences were noted between the patients treated with ranibizumab and aflibercept (92.1 days vs 95.9 days). This is similar to another retrospective US study in patients with DME treated with bevacizumab (3 months) [34].

The time between consecutive treatments was considerably large for patients in this study (nAMD: 73.6 days; DME: 80.5 days) compared with the recommended dosing posology for ranibizumab, which states that treatment should be initiated with 1 injection per month until maximum visual acuity is achieved and/or no signs of disease activity are observed. In patients with nAMD and DME, 3 or more consecutive monthly injections may be needed initially [29]. Aflibercept treatment in patients with nAMD is usually initiated with 1 injection per month for 3 consecutive doses, and the treatment interval is then extended to 2 months. Aflibercept treatment of patients with DME is initiated with 1 injection per month for 5 consecutive doses, followed by 1 injection every 2 months [30].

The dosing interval was lower for ranibizumab compared with aflibercept in patients with nAMD (53.6 days vs 87.4 days), whereas a similar dosing interval was observed between ranibizumab and aflibercept in patients with DME (77.9 days vs 82.5 days, respectively). In general, the dosing interval was larger than that reported by the US claims database study (ranibizumab: 51 days; aflibercept: 54 days) [27]. The dosing interval depended on the treatment regimen chosen: PRN vs treat and extend (T&E). The larger dosing interval could be owing to decreased patient compliance.

Most patients with nAMD or DME received treatment in other settings, which include visit with a daycare bed; during national screening, new visa screening, renewal visa screening; at home, assisted living facility, mobile unit; or ambulance assistance via air/water. It is recommended that anti-VEGF injections are usually administered in outpatient settings with proper infection control [35, 36]. In the UAE, treatment is usually administered in a clean and sterilized environment, irrespective of the treatment setting.

A small number of patients switched anti-VEGF therapy (10.9%–13.5%). The reasons for switching treatment could be an unsatisfactory response, economic considerations, or insurance decisions [37]. However, compared with the overall cohort, patients who switched their anti-VEGF treatment had a shorter interval between consecutive injections (overall vs switchers: nAMD, 73.6 days vs 47.8 days; DME, 80.5 days vs 70.3 days, respectively). Most of the switches were observed in the ranibizumab cohort in both nAMD and DME groups. This might be because ranibizumab has been available for a longer time than aflibercept and clinicians tend to switch to a newer drug if visual outcomes do not improve. This finding is similar to that of the *FRB*! study that reported more switches from ranibizumab to aflibercept than vice versa [38].

Diabetes was observed to be the most common comorbidity (83.8%) in patients with nAMD and DME. Diabetes has been identified as an independent risk factor for AMD, based on a meta-analysis that assessed the association between diabetes and AMD [39]. Furthermore, DME is caused by DR, a complication of diabetes; therefore, a diabetes diagnosis was expected [40].

In the potential bilateral patients, 42.6% of patients did not receive any OAD prescription, while 65.6% of the patients did not have an insulin prescription. This could be owing to the use of traditional and alternative medicine in the UAE [41]. On the contrary, medication adherence to OAD treatment was high in patients with nAMD and DME. This could be owing to the rising awareness and access to educational programs in the UAE, which have been shown to increase adherence in patients with diabetes [42].

The use of medical claims data not designed for research could have increased the chances of bias in this study. The non-availability of the correct International Classification of Diseases codes is another limitation. Medical claims databases may not capture patient services received through out-of-pocket transactions. Since <0.1% of the total claims come from the public sector, it is possible that patients captured in the DRWD may be different from the general Dubai patient population, leading to some degree of selection bias. A larger sample size would have added strength to the study’s observations. In addition, as the current analysis focuses on the real-world anti-VEGF treatment patterns, we did not report on safety data, which are relatively well-known and extensively documented in previous literature. It is also likely that a patient received interim care with other providers, and, in reality, the mean number of injections may be slightly higher than the data reported in this study.

This is the first report on practice patterns from the Middle East including medication adherence and general health status in anti-VEGF recipients. Our study had a systematic selection criteria, good insurance profiling, careful follow-up, and accurately collected data from the DRWD database. Dubai is the most populated emirate in the UAE. The current study provides a critical understanding on the utilization of anti-VEGF treatments among the private healthcare sectors in Dubai. The findings of the current study are similar to the multicenter LUMINOUS study, which observed that patients with nAMD and DME are under-treated in real-life settings [24, 43].

## Conclusions

This real-world study provides an initial understanding of anti-VEGF treatment patterns and characterization of demographic and clinical profiles of patients with nAMD and DME in the Middle East. Anti-VEGF agents were administered infrequently in patients with nAMD and DME. The dosing intervals were also considerably large. The sub-optimal anti-VEGF treatment in patients with nAMD and DME signals an unmet need for better treatment management in real-world settings. Further studies on anti-VEGF treatment patterns in real-world settings in the UAE are needed.

## Supporting information

S1 Appendix Exclusion criteria. (DOCX)

S2 Appendix Study assessments. (DOCX)

S3 Appendix Feasibility analysis prior to screening of patients. (DOCX)

S1 Table Oral/insulin treatment patterns in the follow-up period for nAMD and DME potential bilateral patients. (DOCX)

S1 File Study aggregated data. (XLSX)
