# Supplementary material for: Treatment patterns in patients with age-related macular degeneration and diabetic macular edema: A real-world claims analysis in Dubai
Source: PLoS One. 2021 Jul 13;16(7):e0254569. doi: 10.1371/journal.pone.0254569 (PMC8277020; doi:10.1371/journal.pone.0254569)
Supplement: S1 Appendix — (DOCX) [file pone.0254569.s001.docx]

### S1 Appendix. Exclusion criteria

Patients were excluded if they were known to have received ranibizumab or aflibercept before entering the study or if there was a gap of <21 days between 2 consecutive visits for anti-vascular endothelial growth factor (VEGF) treatment or if they received multiple injections of the anti-VEGF agent on any particular day during the follow-up. This served to exclude patients who could potentially have a bilateral condition at baseline, based on the receipt of consecutive injections earlier than the recommended frequency. Excluded bilateral patients were studied separately for the exploratory objectives related to the use of oral anti-diabetic drugs and insulin.
