# Supplementary material for: Treatment patterns in patients with age-related macular degeneration and diabetic macular edema: A real-world claims analysis in Dubai
Source: PLoS One. 2021 Jul 13;16(7):e0254569. doi: 10.1371/journal.pone.0254569 (PMC8277020; doi:10.1371/journal.pone.0254569)
Supplement: S2 Appendix — (DOCX) [file pone.0254569.s002.docx]

### S2 Appendix. Study assessments The neovascular age-related macular degeneration (nAMD)/diabetic macular edema (DME) diagnosis codes available in the database indicated the presence of disease. However, the last digit of the International Classification of Diseases, 10th Revision (ICD-10) diagnosis code indicating the eye(s) with the disease (right/left/bilateral) was not available. Thus, the patient with a diagnosis of nAMD/DME was the unit of the analysis and not the eyes. The medical claims were coded using the ICD-10-Clinical Modification (ICD-10-CM), Current Procedural Terminology (CPT 4). All patient identifiers were protected according to the Health Insurance Portability and Accountability Act. Electronic entry obviated the need for duplicative, time-consuming manual entry. Patient identifiers were stripped out completely. On this basis, formal ethics approval was not required. Patients who switched from their primary anti-VEGF therapy to a different anti-VEGF therapy were considered to have switched therapy. Adherence was measured using proportion of days covered (PDC) defined as the total number of days supplied for a patient’s prescriptions for the primary therapy during the follow-up period divided by the number of days in the follow-up period. If a patient refilled a prescription early, any overlapping days with the earlier refill were counted only once. Although potential bilateral patients were excluded from the final study population, they were studied separately for this exploratory objective.

The injection visits/settings were classified as: Emergency department visits/settings, inpatient visits/settings, outpatient visits/settings, or other visits/settings depending on the treatment during the follow-up period. The definitions of the treatment settings are as follows: (1) emergency department visits: a visit where emergency room (ER) service was utilized, with or without inpatient bed; (2) inpatient visits: a visit where inpatient bed was utilized, without the use of the ER; (3) outpatient visits: a visit without any use of an inpatient bed or ER; (4) other settings/visits: a visit with a daycare bed; during national screening, new visa screening, renewal visa screening; at home, assisted living facility, mobile unit; or ambulance assistance via air/water.
