# Supplementary material for: Treatment patterns in patients with age-related macular degeneration and diabetic macular edema: A real-world claims analysis in Dubai
Source: PLoS One. 2021 Jul 13;16(7):e0254569. doi: 10.1371/journal.pone.0254569 (PMC8277020; doi:10.1371/journal.pone.0254569)
Supplement: S3 Appendix — (DOCX) [file pone.0254569.s003.docx]

**S3 Appendix. Feasibility analysis prior to the screening of patients**
A feasibility analysis showed the presence of 4,723 and 300 patients in the database with first diagnosis claims for DME and nAMD, respectively, from July 2014 to April 2017 (with an overlap of 4 patients who had a diagnosis for both conditions). Of the 4,723 patients with DME, 333 (7.1%) and 306 (6.5%) had a prescription for ranibizumab and aflibercept, respectively, during the 360-day post-injection period. Similarly, of the 300 patients with nAMD, the number of patients with a prescription for ranibizumab and aflibercept during the 360-day post-injection period was 73 (24.3%) for each. Applying the criterion of a <21-day gap between the 2 primary anti-VEGF injections, 132 patients were excluded.
