# Supplementary material for: Treatment patterns in patients with age-related macular degeneration and diabetic macular edema: A real-world claims analysis in Dubai
Source: PLoS One. 2021 Jul 13;16(7):e0254569. doi: 10.1371/journal.pone.0254569 (PMC8277020; doi:10.1371/journal.pone.0254569)
Supplement: S1 Table — (DOCX) [file pone.0254569.s004.docx]

**S1 Table.** **Oral/insulin treatment patterns in the follow-up period for nAMD and DME potential bilateral patients**

| **Criteria** | **Overall** | **nAMD** | **DME** |
| --- | --- | --- | --- |
| **Number of patients with 2 consecutive visits for primary anti-VEGF treatment within a period of 21 days, N (%)** | **61 (100.0)** | **4 (6.6)** | **57 (93.4)** |
| Number of oral antidiabetic prescriptions during follow-up, n (%) | | | |
| 0 | 26 (42.6) | 3 (75.0) | 23 (40.4) |
| 1 | 5 (8.2) | 0 (0.0) | 5 (8.8) |
| 2 | 2 (3.3) | 0 (0.0) | 2 (3.5) |
| 3 | 4 (6.6) | 0 (0.0) | 4 (7.0) |
| 4 | 5 (8.2) | 0 (0.0) | 5 (8.8) |
| 5+ | 19 (31.1) | 1 (25.0) | 18 (31.6) |
| Number of insulin prescriptions during follow-up, n (%) | | | |
| 0 | 40 (65.6) | 4 (100.0) | 36 (63.2) |
| 1 | 1 (1.6) | 0 (0.0) | 1 (1.8) |
| 2 | 1 (1.6) | 0 (0.0) | 1 (1.8) |
| 3 | 3 (4.9) | 0 (0.0) | 3 (5.3) |
| 4 | 2 (3.3) | 0 (0.0) | 2 (3.5) |
| 5+ | 14 (23.0) | 0 (0.0) | 14 (24.6) |
| **Number of patients with multiple injections of primary anti-VEGF on any particular day during the follow-up** | **105 (100.0)** | **16 (15.2)** | **89 (84.8)** |
| Number of OAD prescriptions during follow-up | | | |
| 0 | 58 (55.2) | 11 (68.8) | 47 (52.8) |
| 1 | 4 (3.8) | 1 (6.3) | 3 (3.4) |
| 2 | 5 (4.8) | 0 (0.0) | 5 (5.6) |
| 3 | 3 (2.9) | 0 (0.0) | 3 (3.4) |
| 4 | 3 (2.9) | 0 (0.0) | 3 (3.4) |
| 5+ | 32 (30.5) | 4 (25.0) | 28 (31.5) |
| Number of insulin prescriptions during follow-up | | | |
| 0 | 72 (68.6) | 15 (93.8) | 57 (64.0) |
| 1 | 11 (10.5) | 1 (6.3) | 10 (11.2) |
| 2 | 2 (1.9) | 0 (0.0) | 2 (2.2) |
| 3 | 1 (1.0) | 0 (0.0) | 1 (1.1) |
| 4 | 0 (0.0) | 0 (0.0) | 0 (0.0) |
| 5+ | 19 (18.1) | 0 (0.0) | 19 (21.3) |

DME, diabetic macular edema; N, total number of patients; n, number of patients; nAMD, neovascular age-related macular degeneration; SD, standard deviation; VEGF, vascular endothelial growth factor.
